# Antimicrobial Effect of Asiatic Acid Against *Clostridium difficile* Is Associated With Disruption of Membrane Permeability

**DOI:** 10.3389/fmicb.2018.02125

**Published:** 2018-09-07

**Authors:** Phurt Harnvoravongchai, Surang Chankhamhaengdecha, Puey Ounjai, Sombat Singhakaew, Kanpong Boonthaworn, Tavan Janvilisri

**Affiliations:** ^1^Department of Biology, Faculty of Science, Mahidol University, Bangkok, Thailand; ^2^Department of Biochemistry, Faculty of Science, Mahidol University, Bangkok, Thailand

**Keywords:** *Clostridium difficile*, asiatic acid, antimicrobials, drug resistance, herb

## Abstract

Antibiotic resistance is a major concern in *Clostridium difficile*, the causative agent of antibiotic-associated diarrhea. Reduced susceptibility to first- and second-line agents is widespread, therefore various attempts have been made to seek alternative preventive and therapeutic strategies against this pathogen. In this work, the antimicrobial properties of asiatic acid were evaluated against *C. difficile*. Asiatic acid displayed substantial inhibitory effects on 19 *C. difficile* isolates collected from different sources with minimal inhibitory concentrations ranging from 10 to 20 μg/ml. Time kill analysis and minimal bactericidal concentration revealed potential bactericidal activity of this compound. Asiatic acid induced membrane damages and alterations in morphological ultrastructure in *C. difficile*, thereby causing the leakage of intracellular substances. Moreover, asiatic acid also displayed an inhibitory effect on cell motility, but did not interfere with biofilm formation and spore germination. Analysis of drug combination showed no synergistic effect between asiatic acid and vancomycin/metronidazole. Altogether, asiatic acid exhibited strong antimicrobial activity against vegetative cells and could serve as an alternative resource for tackling *C. difficile*.

## Introduction

*Clostridium difficile* infection (CDI) is considered as a major leading cause of infectious diarrhea among hospitalized patients. Over the past decades, there has been a progressive increase in the prevalence and mortality of CDI cases worldwide (Martin et al., 2016). Although patients with CDI may develop the disease from hospitals, potential sources of CDI in humans may include domestic and farm animals since an overlap between isolates from humans and animals has been demonstrated (Janvilisri et al., 2009). As a consequence of long-term antibiotic use, normal gastrointestinal biota is disrupted, allowing scarce population of *C. difficile* to overgrow and colonize in the gastrointestinal tract. The pathogenicity of *C. difficile* depends mostly on the toxins A and B. Both toxins induce the disruption of tight junctions of colonic epithelial cells, causing various symptoms ranging from mild diarrhea to severe pseudomembranous colitis (Voth and Ballard, 2005; Kuehne et al., 2010). Vancomycin and metronidazole are normally prescribed for the patients with CDI according to the clinical guideline, however, 25% of the cases continue to suffer from recurrence. In recent years, significant reduction in the susceptibility of *C. difficile* against vancomycin and metronidazole has been demonstrated in clinical isolates, causing treatment failure for CDI (Peláez et al., 2002, 2008; Freeman et al., 2010; Tickler et al., 2014). This could potentially be due to the fact that the *C. difficile* develops defensive mechanisms against these prescribed drugs (Harnvoravongchai et al., 2017; Ngernsombat et al., 2017). Although alternative approaches such as fecal transplantation and phage therapy have been introduced for treatment of CDI, however, limitations due to the immunological concern has been addressed (Bakken et al., 2011; Brandt et al., 2012; Sangster et al., 2014). Hence, novel therapeutic options are still in a critical demand. At present, herbs and natural products have regained tremendous attention to be used as promising alternatives for the treatment of bacterial infection. In fact, various active natural extracts against *C. difficile* have been reported, however, the identification of bioactive lead compounds and characterization of their mode of action are still lacking (Aljarallah, 2016; Finegold et al., 2018). Asiatic acid (AA) is a pentacyclic triterpenoid derived from a tropical plant *Centella asiatica*, which has been widely used in traditional remedy in Asia. AA has been shown to exhibit the beneficial effects not only for anti-cancer and neuroprotective activities (Krishnamurthy et al., 2009; Wu et al., 2017), but also displays antimicrobial activity against certain Gram-positive and Gram-negative pathogenic bacteria (Djoukeng et al., 2005; Wong et al., 2011; Liu et al., 2015). However, there is still no report regarding the effect of AA against *C. difficile.* This study therefore aims to investigate the potential inhibitory effect and the mode of action of AA against *C. difficile.*

## Materials and Methods

### Bacterial Strains, Growth Condition and Chemicals

**FIGURE 1:**
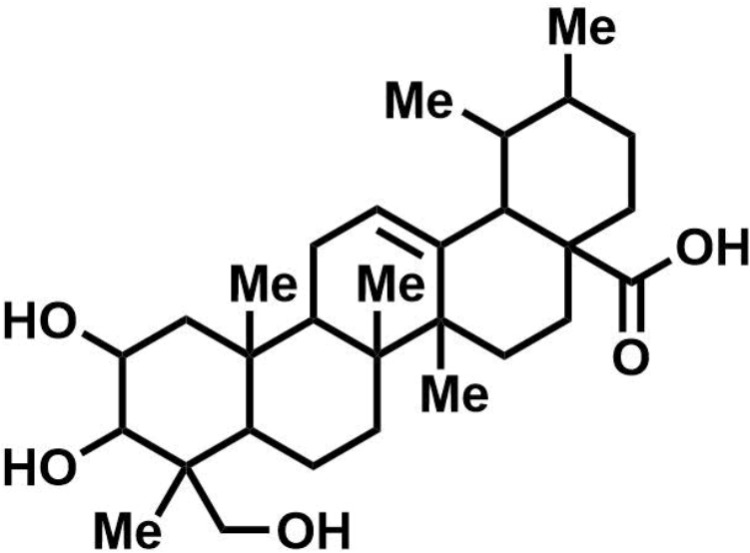
Chemical structure of asiatic acid. Figure was created using ChemDraw version 17.0.0.206 (121). Active code: 683F-0185-F588-C2F2.

*Clostridium difficile* strains 630 and R20291 were kindly provided as a gift from Prof. Nigel Minton, University of Nottingham. Human isolates (RA) of *C. difficile* were previously isolated from diarrheal patients admitted to Ramathibodi Hospital, Thailand during 2010–2011 (Chankhamhaengdecha et al., 2013). The *C. difficile* NIH isolates were obtained from the National Institute of Health (NIH), Thailand. Food and animal *C. difficile* isolates were previously obtained (Ojha et al., 2016). All isolates were cultivated in brain heart infusion (BHI) medium at 37°C under anaerobic condition, unless otherwise mentioned. Asiatic acid with the purity of 97% was purchased from Sigma-Aldrich (St. Louis, MO, United States). The structure of AA is shown in **Figure 1**. The stock AA solution was prepared at 10 mg/ml in dimethyl sulfoxide (DMSO).

### Determination of Minimal Inhibitory Concentrations and Minimal Bactericidal Concentrations

Minimal inhibitory concentrations (MICs) were determined by broth dilution method. One percent of overnight bacterial culture was transferred into pre-reduced BHI broth, followed by incubation at 37°C for 8–10 h to avoid the cells entering spore stage. Cell density was diluted to the final inoculum of approximately 3 × 10^5^ CFU/ml in 96 well-plate containing antimicrobial agents. Following 48 h of incubation, optical density at 600 nm was measured using a microplate reader (Tecan). To determine the minimal bactericidal concentrations (MBCs), cell suspensions in 96 well-plates used for MIC test were replicated onto BHI agar. Following the incubation at 37°C under anaerobic condition for 48 h, further bacterial growth on the agar was visually observed.

### Time-Kill Analysis

The starter was prepared by growing *C. difficile* strains 630 and R20291 culture overnight. The inoculum was done by transferring one percent of starter culture into BHI broth and incubated for 8-10 h to avoid cells entering spore stage. Time kill assays were carried out with the initial inoculum at approximately 10^8^ CFU/ml. Bacterial growth kinetics was followed against different concentrations of AA. The ratio of the absorbance at 600 nm measured at the time *t* and time zero was plotted against time.

### Fluorescent Microscopic Analysis

*Clostridium difficile* strain R20291 suspension was prepared as described above. Cell suspension was treated with 20 μg/ml AA for 0, 2, 4 and 6 h. The samples were then washed, mounted on glass slides, and fixed with methanol. To distinguish live and dead bacterial cells, fixed samples were stained with 500 mM propidium iodide (PI), washed and subsequently counterstained with 1 μg/ml Hoechst 33342. Each sample was visualized and analyzed under FV3000 confocal laser scanning microscope (Olympus). To investigate the cell permeability, *C. difficile* cells were treated with 20 μg/ml AA for 0, 30, 60, and 120 min. The samples then were processed as mentioned previously. After the cells were fixed, each sample was stained with 4′,6-diamidino-2-phenylindole (DAPI), washed and subsequently stained with membrane dye, FM 4-64 (Life technologies). Images were acquired in confocal laser scanning microscope LSM800 with airyscans (Zeiss).

### Determination of Protein and Nucleic Acid Leakage

Five ml of *C. difficile* strain R20291 cells at an optical density of 1.0 were harvested and resuspended in pre-reduced phosphate buffer saline (PBS, pH 7.4). AA was added to a final concentration of 40 μg/ml, the solution was then incubated at 37°C for 6 h under anaerobic condition. Cell debris and pellet were removed by centrifugation, and soluble fraction was retrieved for protein determination using Bradford assay and visualized on SDS-PAGE. For the measurement of nucleic acid leakage, total nucleic acids were purified from the soluble fraction by phenol-chloroform extraction, followed by ethanol precipitation. Quantification of the amount of nucleic acid was monitored by spectrophotometer and further verified by agarose gel electrophoresis.

### Scanning Electron Microscopy

The effect of AA on cell morphology and ultrastructure of *C. difficile* strains 630 and R20291 was observed by scanning electron microscopy (SEM). *C. difficile* suspension was incubated with AA at 37°C for 6 h, and the suspension was collected and resuspended in PBS pH 7.4. The bacterial cells were fixed with the buffer containing 2.5% glutaraldehyde and 4.0% paraformaldehyde for 4 h, followed by post fixation with a cross-linking reagent, 1.0% osmium tetraoxide, for 1 h. The samples were then dehydrated with a series of ethanol, critical point dried through carbon dioxide, followed by sputter coating with platinum-palladium. The specimens were visualized under Hitachi 2500 scanning electron microscope.

### Biofilm Formation and Degradation

One percent of *C. difficile* strain R20291 suspension (Abs_600_ of 0.5) was inoculated into 16 × 150 mm screw capped glass culture tube, containing 5 ml BHI supplemented with 0.1 M glucose. The culture was grown for 5 days to allow biofilm development. Different concentrations of AA were added into the bacterial biofilm, followed by further incubation for 24 h. Remained biofilm was measured with crystal violet staining as described previously with slight modifications (Dawson et al., 2012). The samples were washed twice with PBS to remove unbound cells, subsequently stained with 0.2% crystal violet for 30 min, and washed twice with PBS. Absolute methanol was used to extract crystal violet from the biofilm and the solution was monitored at the absorbance of 570 nm.

### Evaluation of Bacterial Motility

The effect of AA on swimming motility of *C. difficile* strain 630 was determined on soft agar. Two microliter of *C. difficile* culture was stabbed into pre-reduced 0.4% agar BHI medium with different concentrations of AA, followed by the incubation at 37°C for 48 h under anaerobic condition. Swimming motility was quantified by measuring diameter of the spot on soft agar.

### Spore Preparation and Germination Kinetics

Overnight culture of *C. difficile* strains 630 and R20291 was plated onto 70:30 sporulation medium (Fimlaid et al., 2013), followed by the incubation for 3–4 days. Spores were then harvested as previously described (Ojha et al., 2016). Briefly, sporulation induced lawns were collected in distilled water using sterile scarper and washed in distilled water for 5 times. The suspensions were treated with 0.3 mg/ml proteinase K for 1 h, then subjected to heat at 65°C for 1 h to allow efficient inactivation of vegetative cells. Spores were additionally washed with distilled water for 5 times to remove cell debris. Phase contrast microscopy was used to examine the spore purity and spore viability was checked on the agar plate, before being stored at room temperature. To investigate the germination kinetics, germination assay was evaluated as previously described with a few modifications (Bhattacharjee et al., 2015). Spore suspensions were heat-activated at 65°C for 30 min, then subsequently chilled on ice. Activated spores were diluted in BHI supplemented with 0.1% taurocholate. Spore germination was tracked by monitoring the loss of Abs_600_ at 1 min interval for 1 h. The ratio of the Abs_600_ at the time *t* and time zero was plotted against time.

### Drug Combination Assay

Bacterial susceptibility to the combination of AA and either vancomycin or metronidazole was tested by checkerboard assay (Magi et al., 2015). Two-fold serial dilutions of AA at the final concentrations ranging from 0.625 to 40 μg/ml were mixed with either vancomycin at a range of 0.25–16 μg/ml or metronidazole at a range of 0.03125–2 μg/ml in 96-well plates. Approximately 3 × 10^5^ CFU/ml of *C. difficile* strains 630 and R20291 were inoculated, followed by the incubation at 37°C for 48 h under anaerobic condition. To determine the drug combination effect, fractional inhibitory concentration (FIC) index was calculated according to the equation: FIC index = FIC_A_ + FIC_B_, where FIC_A_ = MIC_A+B_/MIC_A_ and FIC_B_ = MIC_B+A_/MIC_B_. MIC_A+B_ is the MIC of compound A in the combination with compound B and *vice versa* for MIC_B+A_, whereas MIC_A_ or MIC_B_ are the MIC of the compound alone. The combination effect is defined as synergy when FIC ≤ 0.5, additive when 0.5 < FIC ≤ 1.0, no interaction when 1.0 < FIC ≤ 4.0, and antagonism when FIC > 4.0 (Páez et al., 2013).

### Statistical Analysis

Unless indicated otherwise, all statistical analyses were based on at least 3 independent experiments. The statistical analyses were performed using Student’s *t*-test with a 95% confidence interval for the sample mean. The *p*-value less than 0.05 indicates statistically significant difference.

## Results

### Asiatic Acid Inhibits the Growth of *C. difficile*

An inhibitory effect of AA on 19 *C. difficile* strains isolated from different sources was investigated by determining the MIC values. The result of MICs of AA and recommended antibiotics used for CDI treatment including vancomycin and metronidazole were summarized in **Table 1**. AA exhibited substantial inhibitory effect against *C. difficile* strains with the MIC value of 10.0 μg/ml, excepted for human isolates 630 and RA156, whose MIC values were 20.0 μg/ml. On the contrary, a broad range of MICs of vancomycin and metronidazole against the tested *C. difficile* strains was evident. The MICs of vancomycin ranged from 1.0–8.0 μg/ml, while the MICs of metronidazole were from 0.5–16.0 μg/ml. MBC assays were concomitantly performed to demonstrate the bactericidal effect of AA on *C. difficile*. According to **Table 1**, MBCs of AA against *C. difficile* was equal to 10.0 μg/ml, except for the strains 630, R20291 and RA156, which MBC values were shifted up to 20.0 μg/ml.

**Table 1:** Antimicrobial activity of asiatic acid, vancomycin, and metronidazole against *C. difficile* isolated from different sources.

Strains	MIC (μg/ml)			MBC (μg/ml)	Sources
	Asiatic acid	Vancomycin	Metronidazole	Asiatic acid	
R20291	10.0	1.0	0.5	10.0–20.0	Human
630	10.0–20.0	2.0	0.5	10.0–20.0	Human
Fd001	10.0	8.0	16.0	10.0	Food
Fd002	10.0	4.0	8.0	10.0	Food
Fd003	10.0	2.0	8.0	10.0	Food
Bv001	10.0	2.0	8.0	10.0	Animal
Bv002	10.0	2.0	8.0	10.0	Animal
Sw001	10.0	4.0	8.0	10.0	Animal
Ct001	10.0	2.0	8.0	10.0	Animal
RA037	10.0	4.0	2.0	10.0	Human
RA044	10.0	1.0	0.5	10.0	Human
RA156	10.0–20.0	1.0	1.0	10.0–20.0	Human
RA376	10.0	1.0	1.0	10.0	Human
NIH001	10.0	1.0	1.0	10.0	Human
NIH011	10.0	2.0	1.0	10.0	Human
NIH017	10.0	8.0	4.0	10.0	Human
NIH028	10.0	8.0	16.0	10.0	Human
NIH042	10.0	4.0	16.0	10.0	Human
NIH031	10.0	2.0	16.0	10.0	Human

*At least 3 biological replicates were performed to ensure the reproducibility*.

**FIGURE 2:**
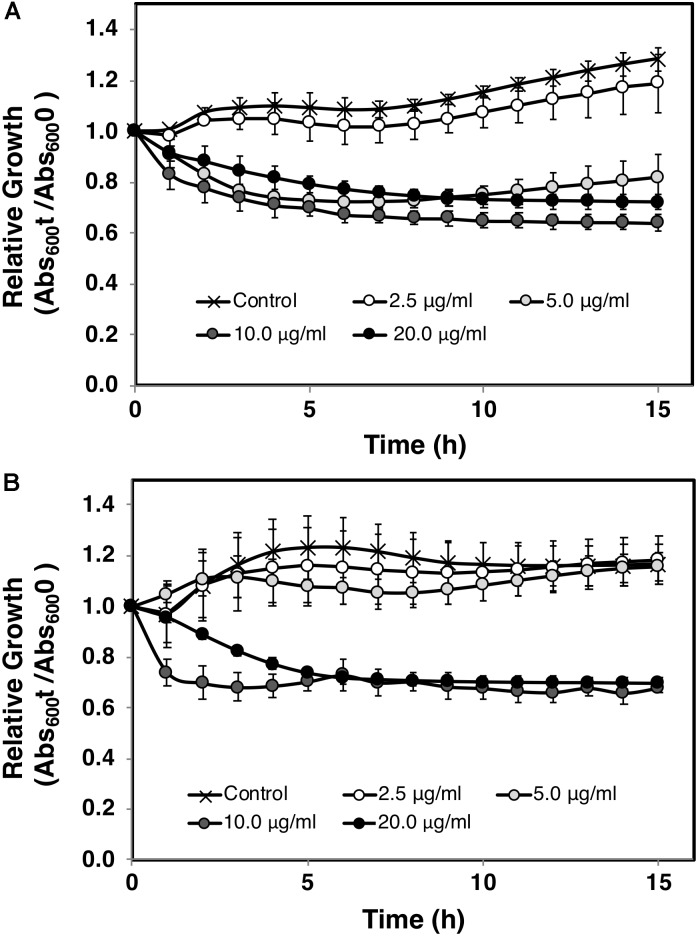
Time kill curve of *C. difficile* treated with asiatic acid. *C. difficile* strains **(A)** R20291 and **(B)** 630 were grown in BHI broth medium with various concentrations of asiatic acid. Growth kinetics was followed for 15 h at 1 h interval. 1% DMSO was added to the control sample. Mean values of at least 3 independent measurements (± standard error) are plotted.

**FIGURE 3:**
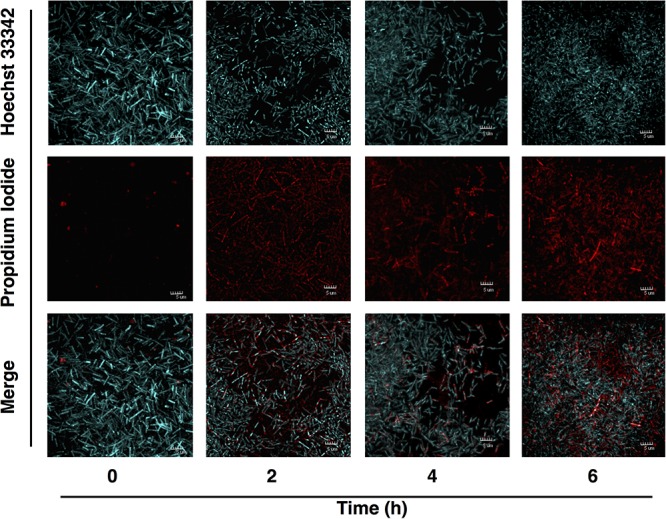
Viability of *C. difficile* following treatment with asiatic acid. Fluorescent images were captured after *C. difficile* strain R20291 was exposed to AA at 0, 2, 4, and 6 h. Blue-stained bacteria (Hoechst 33342) represented live cell, while red-stained bacteria (propidium iodide) represented dead cell.

The effect of AA on *C. difficile* growth was assessed using *in vitro* time kill experiments. The relative growth as measured by Abs_600_ at the time t compared to *t* = 0 was plotted against times. An apparent reduction of bacterial viability was seen as indicated by the decline of the slope (**Figure 2**). In agreement with MIC/MBC data, the exposure to AA at the concentrations of 10 and 20 μg/ml totally inhibited the growth of both *C. difficile* strains 630 and R20291. Bactericidal effect of AA was further validated by live and dead cell assays using PI/Hoechst 33342 staining and fluorescent microscopy. PI is a membrane impermeant dye, while Hoechst 33342 exhibits membrane permeability in bacterial cells, therefore only dead cells accumulate and display red intensity. As shown in **Figure 3**, the levels of red intensity increased in a time-dependent manner, implying the higher population of dead cells over the exposure time. These results were strongly consistent with the bactericidal activity indicated in the time kill curve.

### Asiatic Acid Disrupts *C. difficile* Membrane Permeability

**FIGURE 4:**
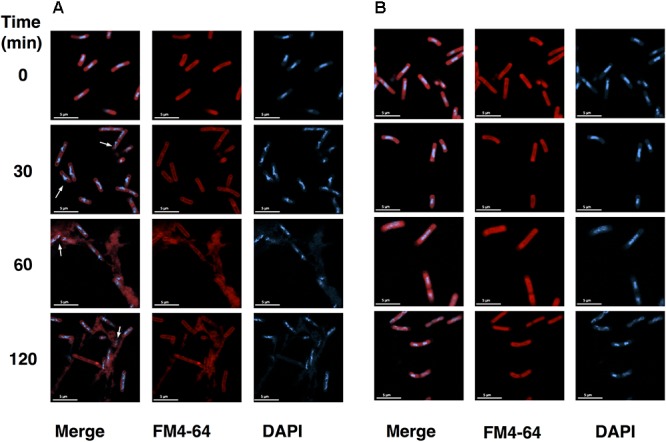
*C. difficile* membrane integrity is compromised in the presence of asiatic acid. Fluorescence microscopic images of **(A)** *C. difficile* strain R20291 treated with 20 μg/ml AA at each time interval and **(B)** untreated samples stained with membrane dye FM4-64 (Red) and DNA dye DAPI (Blue). Red fluorescence indicates membrane disintegration (permeable membrane). Scale bar: 5 μm.

**FIGURE 5:**
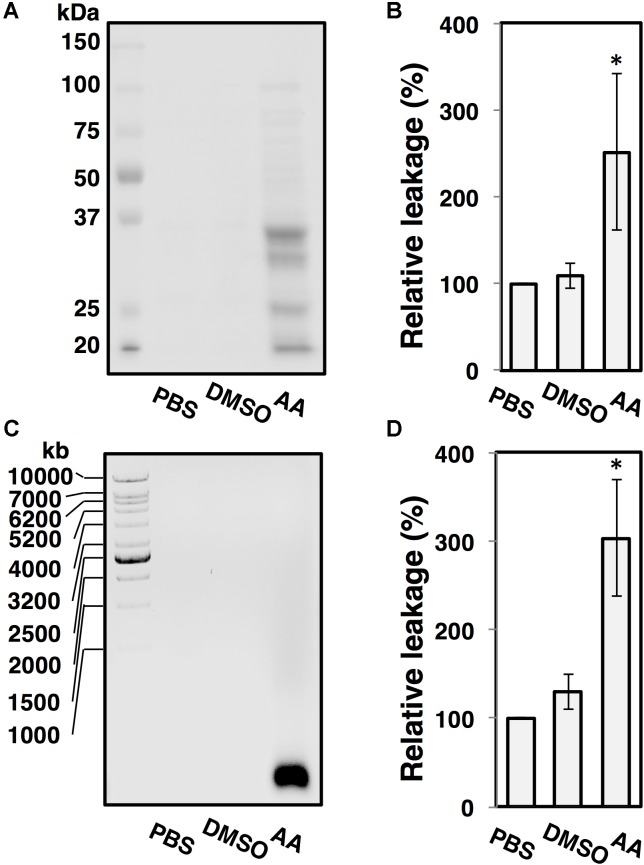
Asiatic acid causes bacterial intracellular component leakage. *C. difficile* strain R20291 grown in BHI medium was anaerobically incubated in PBS buffer, PBS with 1% DMSO and PBS with 40 μg/ml AA. **(A)** Released proteins from the supernatant fractions were analyzed by SDS-PAGE, **(B)** Bar graphs represent the relative protein leakage as determined by Bradford assay, **(C)** Released nucleic acids from the supernatant fractions were analyzed by agarose gel electrophoresis, **(D)** Bar graphs represent the relative nucleic acid leakage as quantified by UV spectrophotometry. At least 3 independent tests were performed to ensure the reproducibility. The error bars and the asterisks represent the standard errors and the statistical significance with the *p*-values < 0.05, respectively.

**FIGURE 6:**
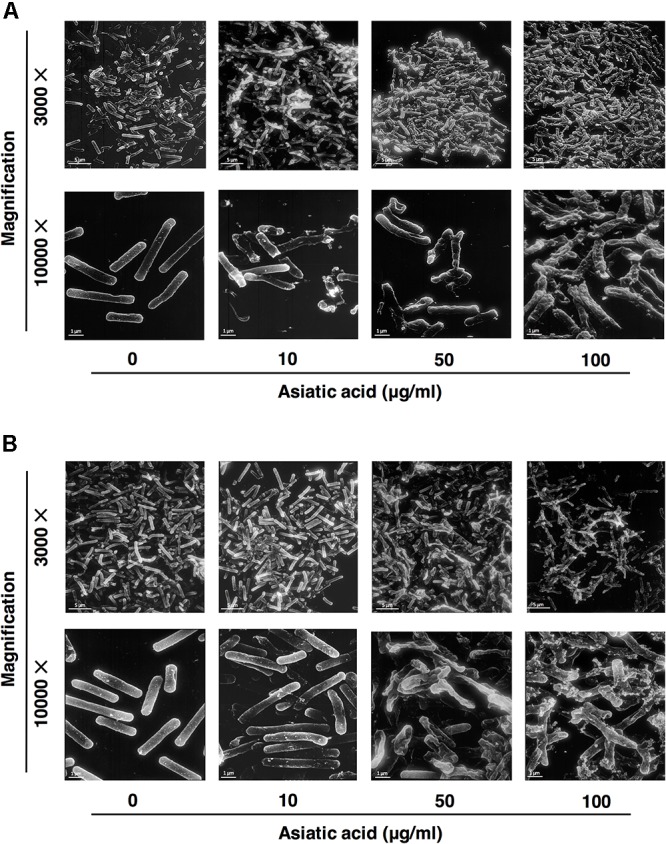
Scanning electron microscopic analysis of asiatic acid-treated *C. difficile* strains **(A)** R20291 and **(B)** 630. Bacterial cells were treated with 10, 50, and 100 μg/ml AA, and were then subjected to SEM. Control (untreated) cells displayed intact smooth surfaces as shown in the left panel. Scale bar: 1, 5 μm.

Changes in bacterial cellular morphology were observed using FM4-64/DAPI staining and fluorescent microscopy. As shown in **Figure 4**, leakage of bacterial DNA was observed in *C. difficile* R20291 following the exposure to AA for 30 min (**Figure 4A**), and the damages to plasma membranes were observed at the exposure time of 60 and 120 min. In contrast, DNA was tightly packed in the intact cells for untreated control condition (**Figure 4B**). The leakage of proteins and nucleic acids in the suspension was also evaluated. Up to 2.5-fold of protein leakage was observed in the cell suspension of *C. difficile* following the AA treatment as shown in **Figures 5A,B**, and the increase was calculated to be statically significant (*p* < 0.05). Similarly, the amount of released nucleic acids in the suspension was significantly elevated to ∼3-fold compared to the untreated controls (**Figures 5C,D**). Alterations in the ultrastructure of *C. difficile* cells following the exposure to AA were observed using SEM analysis (**Figure 6**). While the control cells retained their normal rod-shaped structure with smooth and intact surfaces, the exposure to AA caused damages on the bacterial plasma membrane as indicated by the number of craters on the surface and rupture of cells. The intense of damages was correlated well with the concentration of AA.

### Asiatic Acid Inhibits Cell Motility, but Does Not Interfere Biofilm and Spore Germination

**FIGURE 7:**
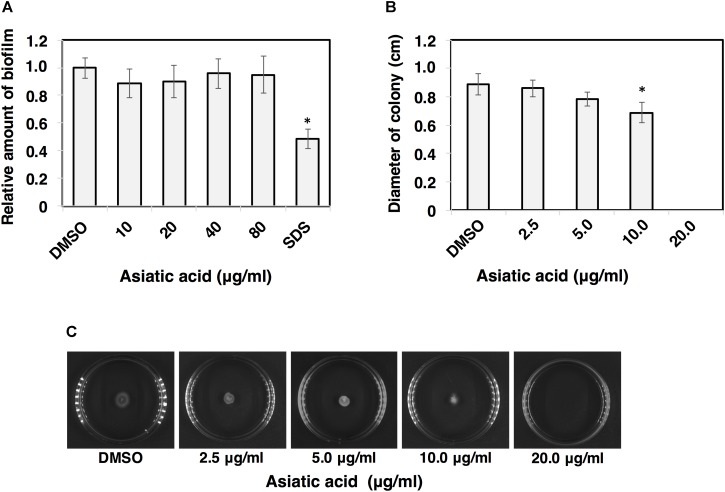
Asiatic acid inhibits *C. difficile* motility, but does not induce biofilm degradation. **(A)** Effect of AA at different concentrations on biofilm degradation in *C. difficile* strain R20291. SDS-treated positive control was included. **(B)** Bar graphs represent migration distance of *C. difficile* strain 630. **(C)** Images represent swimming motility of *C. difficile* strain 630 on BHI soft agar at various concentrations of AA. At least 3 independent experiments were performed. The error bars and the asterisks represent the standard errors and the statistical significance with the *p*-values < 0.05, respectively.

**FIGURE 8:**
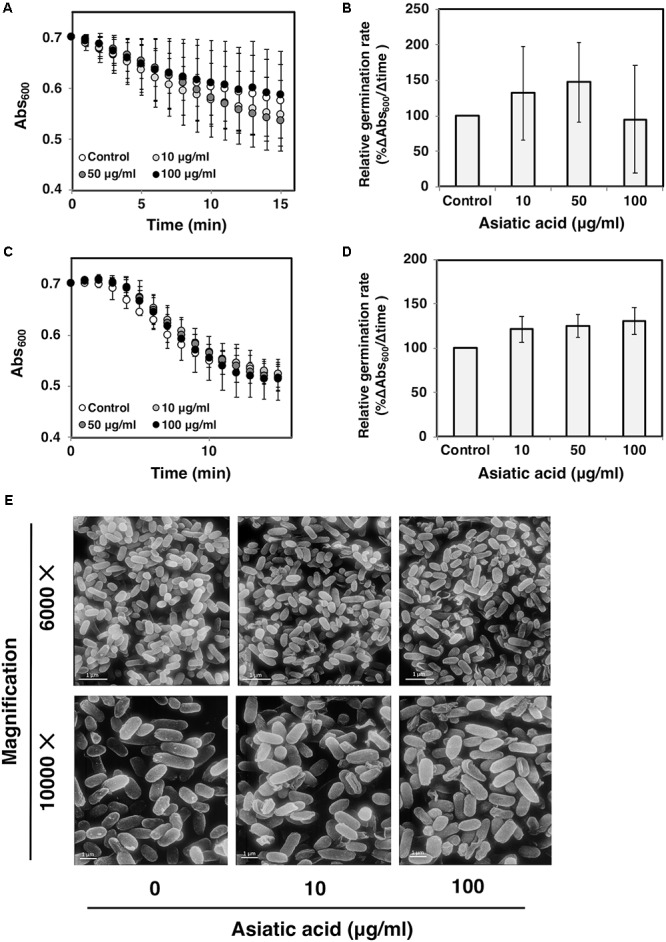
Asiatic acid does not inactivate *C. difficile* spore germination. *C. difficile* spores from strains **(A,B)** R20291 and **(C,D)** 630 were exposed to various concentrations of AA. The germination kinetics was tracked for 15 min at 1 min interval. Bar graphs represent relative germination rate. At least 3 independent tests were performed to ensure the reproducibility. Mean values of at least 3 independent experiments (± standard error) are plotted. **(E)** Scanning electron micrographs of *C. difficile* spores from strain R20291 exposed to 10 and 100 μg/ml AA.

The effects of AA on other virulence factors including biofilm, motility and spore germination were investigated. Ability of *C. difficile* to adhere and form biofilm causes an increase in bacterial virulence (Dapa and Unnikrishnan, 2013). Biofilm of *C. difficile* R20291 was grown and subjected to AA. The results revealed that the amount of biofilm was not affected even upon the exposure of high concentration of AA (80 μg/ml) (**Figure 7A**). For cell motility, the significant reduction in colony diameters of *C. difficile* 630 was observed when the concentration of AA reached 10 μg/ml (*p* < 0.05) (**Figures 7B,C**). It should be noted that no colony formation was seen when the concentration of AA exceeded 10 μg/ml. To determine whether AA could inactivate *C. difficile* spore, purified spores of *C. difficile* strains R20291 and 630 were subjected to AA, and germination kinetics was followed by monitoring the changes in Abs_600_ over a 1 h time period. The relative germination rate was calculated along with the increasing AA concentrations. The results revealed no significant changes in spore germination of both strains at any tested concentrations of AA (**Figures 8A–D**). Despite no effect of AA on spore germination, ultrastructure of C. *difficile* R20291 exhibited a few visible damages on the spore surface following the exposure to 10 and 100 μg/ml AA (**Figure 8E**).

### Asiatic Acid Exhibits No Synergism With Vancomycin and Metronidazole

The synergistic effect of AA and vancomycin/metronidazole was explored using checkerboard method and the isobolograms were plotted (Supplementary Materials). Based on our results, AA exhibited no synergistic effect with both vancomycin and metronidazole as FIC indices were calculated to fall in a range of 1.0 to 1.5, suggesting that AA acted individually with vancomycin or metronidazole.

## Discussion

Prior to the era of antibiotic discovery, folk remedies were widely practiced through the combination of traditional knowledge and implementation of herb medication, health prevention and promotion. Despite the fact that current medication is based on the use of antibiotics, a considerable part of the world population still depends on alternative treatment. In many countries, more than 50% of medical prescriptions rely on traditional medicine, meanwhile 38% of adult patients in the United States preferred herbal medicines as an alternative treatment (Benzie and Wachtel-Galor, 2011). AA, an active constituent mostly present ∼0.5% of dry weight in the leave of *Centella asiatica* (Rafamantanana et al., 2009), has been proved to possess several pharmacological effects including anti-inflammatory, anti-aging and anti-cancer properties (Huang et al., 2011; Tsao and Yin, 2015; Ren et al., 2016). AA also exhibits an antimicrobial activity against certain pathogenic bacteria (Liu et al., 2015). As the current therapy for *C. difficile* relies on antibiotic treatment including vancomycin and metronidazole, and the resistance to both drugs is becoming more pronounced, hence we attempted to explore the potential use of AA as an alternative therapeutic against *C. difficile*.

Susceptibility tests of *C. difficile* with AA showed consistent MIC value among isolates from different sources (10–20 μg/ml). A lower inhibitory concentration of AA against *C. difficile* was observed compared to the previously reported MICs of 24–40 μg/ml on other pathogens (Liu et al., 2015). Variations could be resulted from differences in tested bacterial species and experimental conditions. In parallel, MIC values of vancomycin and metronidazole, the antibiotics recommended in the practical guideline for treatment of CDI, were also determined. The MICs of these two antibiotics varied among tested strains. The human isolate NIH028 exhibited the highest MIC against vancomycin and metronidazole (8.0 and 16.0 μg/ml, respectively) implying the possibility of the resistance development during the antibiotic treatment. The resistance to vancomycin and metronidazole in *C. difficile* strains isolated from the patients in hospitals has been demonstrated (Peláez et al., 2002, 2008; Baines et al., 2008; Spigaglia et al., 2011; Karlowsky et al., 2012). The food isolate Fd001 also exhibited high resistance to both drugs. Because the transmission of *C. difficile* occurs via fecal-oral route, the emergence of antibiotic resistant isolates from food marks an alarm for critical concerning on the transmission of *C. difficile* in contaminated diet (Lund and Peck, 2015). Although higher concentration of AA was required to inhibit certain *C. difficile* isolates, compared to those in vancomycin and metronidazole, however, constant dosage of the compound used for the inhibition allows the determination of precise dose for alternative treatment. MBCs of AA in all tested isolates ranged between 10.0 and 20.0 μg/ml, which do not exceed 4 times of the MICs, indicating the bactericidal activity of the compound. In accordance with the MBC test, time kill kinetics determined in *C. difficile* strains R20291 and 630 demonstrated a decline in bacterial density as a result of AA exposure. *C. difficile* strain R20291 exhibited higher sensitivity to AA compared to the strain 630, which is in agreement with those observed in MIC test. It should be noted that the kinetic studies were followed for 16 h, while MIC determinations were performed for 48 h, therefore the dissimilarity of the susceptibility profiles could be clearly demonstrated between each time intervals rather than at one end point. An increase in the population of dead cells following the exposure to AA was evidently time-dependent, which further supports the role of AA as a bactericidal agent. Prior to the drug administration, cytotoxicity of the compounds against host cells is needed to be evaluated (Tharmalingam et al., 2018). IC_50_ values for AA were reported to be greater than 100 μM (∼50 μg/ml) on human umbilical vein endothelial cells and kidney epithelial Vero cells (Jirasripongpun et al., 2012; Huang et al., 2016), as well as no significant effect of the compound at the same concentration was observed on viability of human gastric mucosa epithelial cells (Jing et al., 2016). Additionally, up to 165 mg/kg of AA was administered to the mice without any signal of side effects (Krishnamurthy et al., 2009). Hence, AA could be a decent therapeutic agent for CDI due to the substantially low MIC against *C. difficile* compared to the cytotoxicity level.

AA is a terpenoid, an organic compound made up of isoprene units, which is usually found in plant essential oils. It has been demonstrated that terpenoids can cause large-scale membrane thinning on lipid bilayer and could therefore exert their antimicrobial properties via a membrane disruption mechanism (Khandelia et al., 2010). Correspondingly, several studies on terpenoids extracted from plants proposed the properties of the compounds to act on the bacterial plasma membrane, which eventually lead to cell dead (Jasmine et al., 2011; Sathya Bama et al., 2012). To determine the potential mode of action of AA on cell permeability, leakage of intracellular compounds was evaluated. The fluorescence microscopic analyses revealed the rapid action of AA on *C. difficile*, which caused the leakage of DNA from the bacterial cells within 30 min, while the shape of cell was still retained. Distorted bacterial membrane with no enclosed DNA was later visualized upon the longer incubation period, in accordance with the amount of released proteins and nucleic acids quantified in the supernatant. Additionally, severe damages at the surface including cell rupture and alterations in cellular morphology as demonstrated in SEM micrographs of both 630 and R20291 strains were consistent with the damages previously observed in other bacteria (Hartmann et al., 2010; Goldbeck et al., 2014; Hong et al., 2015). These data suggested that AA potentially acts on cell membrane and causes substantial membrane rupture on *C. difficile.* As the integrity of bacterial plasma membrane is crucial not only for cell protection but also houses enzymes responsible for cellular processes such as energy production. Thus, bacterial membrane disruption could collapse cellular processes, which in turn affects *C. difficile* survival and growth.

Ability to motile and adhere involves in the development of disease of *C. difficile* (Twine et al., 2009; Dingle et al., 2011; Stevenson et al., 2015). Thus, we assessed the inhibitory effect of AA on the motility of *C. difficile*. The results showed that the motility of *C. difficile* was significantly reduced following the exposure to AA. Noteworthy, even with a concentration below the inhibitory dose, AA could potentially reduce the infection by impairing colonization and adhesion of *C. difficile*. Previous reports showed that certain naturally derived compounds including anthocyanins, could inhibit toxin secretion via transcription repression in a gastrointestinal pathogen, *Helicobacter pylori*, at the sub-inhibitory concentrations (Kim et al., 2012). Further investigations should be addressed for toxin reduction in *C. difficile*. Following *C. difficile* colonization, biofilm formation could be initiated. Bacterial biofilm is an aggregated form of bacterial community, in which each individual cell is compacted and connected through the released extracellular polymeric substances. It has been shown that biofilm formation increases the resistance to vancomycin up to 10 folds in *C. difficile* (Dapa and Unnikrishnan, 2013), reflecting the consequent complication for antibiotic treatment. Although AA exhibited the strong inhibitory effect on *C. difficile* via the disruption of membrane permeability, degrading activity of the compound was not observed when tested against biofilm. Extracellular polymeric substances are usually composed of different biomolecules such as polysaccharides, proteins, DNA and lipids, which serve as a rigid architecture for bacteria. Hence, AA that is supposed to act on lipid membrane, might not be able to penetrate through the barrier of the complicated extracellular matrix of the biofilm.

*Clostridium difficile* spores are highly resistant to harsh conditions including heat and toxic chemicals, and play a critical role in the transmission of the disease. Physical and chemical treatments have been reported to inactivate spores and inhibit their germination (Rutala et al., 1993; Perez et al., 2005; Fawley et al., 2007; Francis et al., 2013; Ojha et al., 2016). Here, we also attempted to examine the effect of AA on spore germination. Although AA exhibited an inhibitory effect against *C. difficile* vegetative cells, however, spore germination did not seem to be significantly influenced by the presence of AA, even at high concentration. On the contrary with the germination data, SEM analysis revealed a detectable spore damage when exposed to 10 μg/ml of AA. It could be explained by the fact that (i) only a few spores were damaged, thus the overall germination rate was not affected, and (ii) spores are encoded by several rigid layers (Paredes-Sabja et al., 2014), therefore the damages at the spore surface may not affect spore germination.

Furthermore, synergism between AA and the first- and second-line agents for CDI including vancomycin and metronidazole was investigated. The FIC values of AA and vancomycin/metronidazole fell into the range of 1.0–1.5, suggesting no synergistic effect between AA and these drugs. This might due to the difference in mode of actions as AA is proposed to disrupt bacterial cell membrane, while vancomycin and metronidazole kill bacteria through the inhibition of cell wall and DNA synthesis, respectively (Huang et al., 2009). Considering no antagonistic effect, CDI patients who have been treated with vancomycin or metronidazole, can be alternatively treated with AA without any contravention.

## Conclusion

Asiatic acid exhibited remarkable antimicrobial activity against *C. difficile* by disrupting permeability of the cell membrane. Nevertheless, the compound was unable to destroy dormant spores or biofilm. Taken into account of the strong inhibition properties of AA and low cytotoxicity level, the compound could be further developed as an alternative treatment to combat CDI.

## Author Contributions

PH and TJ conceived and designed the study. PH, SC, and PO performed the experiments. SS and KB helped with the experimental assays. PH and TJ wrote the paper. TJ supervised the project. All authors have read and approved the manuscript.

## Conflict of Interest Statement

The authors declare that the research was conducted in the absence of any commercial or financial relationships that could be construed as a potential conflict of interest. The reviewer PD and handling Editor declared their shared affiliation.

## Supplementary Material

FIGURE S1 No synergistic effect between asiatic acid and vancomycin/metronidazole. **(A)** Checkerboard test of *C. difficile* strains 630 and R20291 on BHI medium supplemented with the combination of AA and either vancomycin or metronidazole. Shading area represents visible growth. **(B)** Isobolograms of AA plus vancomycin or metronidazole. Dotted lines represent the additive effect of the drug combination (FIC = 1). At least 3 independent tests were performed to ensure the reproducibility.
