# Supplementary figures and images for: Antimicrobial Effect of Asiatic Acid Against Clostridium difficile Is Associated With Disruption of Membrane Permeability
Source: Front Microbiol. 2018 Sep 7;9:2125. doi: 10.3389/fmicb.2018.02125 (PMC6137100; doi:10.3389/fmicb.2018.02125)

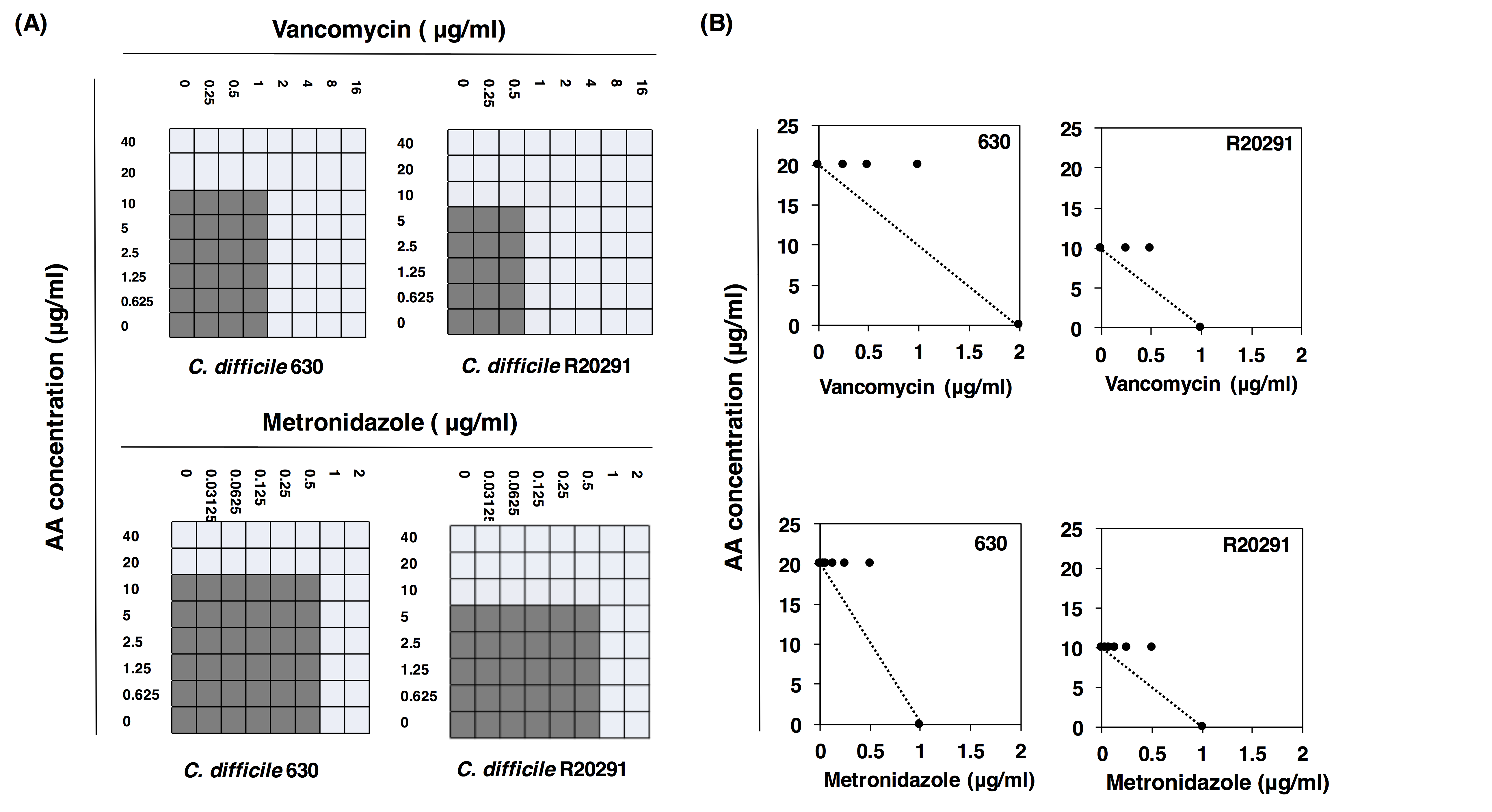

Supplement: FIGURE S1 — No synergistic effect between asiatic acid and vancomycin/metronidazole. (A) Checkerboard test of C. difficile strains 630 and R20291 on BHI medium supplemented with the combination of AA and either vancomycin or metronidazole. Shading area represents visible growth. (B) Isobolograms of AA plus vancomycin or metronidazole. Dotted lines represent the additive effect of the drug combination (FIC = 1). At least 3 independent tests were performed to ensure the reproducibility. [file Image_1.TIFF]
